# Ultra-early endovascular treatment improves prognosis in High grade aneurysmal subarachnoid hemorrhage: A single-center retrospective study

**DOI:** 10.3389/fneur.2022.963624

**Published:** 2022-08-12

**Authors:** Botao Wu, Zhe Huang, Huan Liu, Jiayao He, Yan Ju, Ziwei Chen, Taiwei Zhang, Fuxin Yi

**Affiliations:** ^1^Department of Neurosurgery, The First Affiliated Hospital of Jinzhou Medical University, Jinzhou, China; ^2^Department of Radiology, The First Affiliated Hospital of Jinzhou Medical University, Jinzhou, China; ^3^Department of General Surgery, Zhongshan Hospital of Traditional Chinese Medicine, Guangzhou University of Traditional Chinese Medicine, Zhongshan, China; ^4^Department of Orthopedics, Affiliated Hospital of Chengdu University, Chengdu, China; ^5^Department of Endocrinology, The First Affiliated Hospital of Jinzhou Medical University, Jinzhou, China; ^6^Department of Neurosurgery, The Third Affiliated Hospital of Jinzhou Medical University, Jinzhou, China

**Keywords:** ultra-early, aneurysmal subarachnoid hemorrhage, endovascular treatment, prognosis, high-grade, risk factors

## Abstract

**Background:**

The long-term survival prognosis of patients with high-grade (Hunt-Hess grade IV–V or World Federation of Neurosurgical Societies grade IV–V) aneurysmal subarachnoid hemorrhage (aSAH) is generally poor, and the association between endovascular treatment timing and the prognosis of high-grade aSAH has not been explored in depth. This retrospective cohort study aimed to determine whether endovascular treatment within 24 h of high-grade aSAH is associated with a better prognosis.

**Methods:**

We retrospectively analyzed the clinical data of patients with high-grade aSAH who were admitted to our institution between January 2018 and January 2021. The Modified Rankin Scale score was used to assess the 6-month prognosis of patients. Univariate and multivariate logistic regression analyses were used to identify the factors associated with prognosis. The area under the receiver operating characteristic (ROC) curve was used to assess the model's discriminatory ability.

**Results:**

Eighty-six patients were included in the study. In the multivariate analysis, the timing of endovascular treatment (odds ratio = 7.003 [1.800–27.242], *P* = 0.005) was an independent risk factor for prognosis. The ROC curve showed that the predictive power of the timing of endovascular treatment was 0.744, the best cut-off value was 12.5 h, and the corresponding sensitivity and specificity were 71.4 and 70.5%, respectively. Hydrocephalus (*P* = 0.005) and pulmonary infection (*P* = 0.029) were also associated with prognosis. In addition, cerebrospinal fluid drainage immediately after endovascular treatment had a significant effect on reducing hydrocephalus formation.

**Conclusions:**

Endovascular therapy within 24 h is feasible and improves the prognosis of patients with high-grade aSAH.

## Introduction

Aneurysmal subarachnoid hemorrhage (aSAH) is a disease with an extremely high mortality rate. The higher the aSAH grade, the poorer the prognosis (1–3). High-grade intracranial aneurysms account for 20–30% of ruptured aneurysms, and the mortality rate is as high as 30–60% (4–7). Traditional treatment attitudes have been relatively conservative due to previously reported high mortality rates and poor neurological outcomes in patients with high-grade aSAH (8–10). Currently, the main treatment methods are microsurgical clipping and endovascular coiling (11). The disadvantages of early clipping of high-grade aneurysms include cerebral edema, difficult aneurysm exposure, and a high risk of intraoperative re-bleeding (12). The multicenter randomized International Subarachnoid Aneurysm Trial showed that interventional embolization can improve the prognosis of aneurysm rupture, (13) and early endovascular treatment may be preferred for high-grade aneurysms. Previous studies have also shown that early (within 72 h) endovascular treatment is safer and more effective than delayed endovascular treatment (14–16). However, the concept of ultra-early treatment (within 24 h) in patients with high-grade aSAH is still debated (17–24). Therefore, it is necessary to study the optimal timing for endovascular treatment. The purpose of this single-center retrospective cohort study is to explore the clinical efficacy of ultra-early endovascular treatment and the factors that may affect prognosis, as well as to provide some reference for the timing of endovascular treatment for patients with high-grade intracranial aneurysms.

## Methods

### Patient identification and selection

We reviewed all patients with endovascular treatment between January 2018 to January 2021 in our institution. Inclusion criteria: aged 18–80 years; aSAH diagnosed by computed tomography (CT) or lumbar puncture in the medical center; aneurysm confirmed as the cause of SAH on digital subtraction angiography (DSA), three-dimensional CT angiography, or magnetic resonance angiography, which was the cause of the subarachnoid hemorrhage; endovascular therapy was performed; Hunt-Hess grade IV–V. Exclusion criteria: intracranial aneurysm rupture caused by trauma and unexplained subarachnoid hemorrhage; Hunt-Hess grade ≤ III; microsurgical clipping surgery or conservative treatment; patients lost to follow-up.

### Clinical parameters

The baseline data of the patients were recorded, including gender, age, smoking history, drinking history, hypertension history, diabetes history, coronary heart disease history, aneurysm rupture history, Hunt-Hess grade at admission; aneurysm imaging features such as aneurysm size (maximum diameter and aneurysm neck width), location; and whether stent-assisted or not, postoperative lumbar drainage or external ventricular drain; postoperative complications such as pulmonary infection, intracranial infection, hydrocephalus. The modified Rankin Scale was determined through telephone interviews at 6 months after discharge. The interviewer was blinded to the condition.

The study has been approved by the Ethics Committee of Jinzhou Medical University.

### Outcome assessment

The primary outcome was functional independence, defined modified Rankin Scale (mRS) score of 0–2 (0 = no symptoms at all; 1 = no significant disability despite symptoms and able to carry out all usual duties and activities; 2 = slight disability and unable to carry out all previous activities, but able to look after own affairs without assistance) as good prognosis and 3–6 (3 = moderately disabled and requires some assistance, but no assistance is required for walking; 4 = severely disabled, unable to walk without the assistance of others and unable to take care of the needs of their own body; 5 = extremely severe disability, bedridden, incontinence, and requires ongoing care and attention; 6 = clinical death) as poor prognosis. Functional outcomes were assessed using inpatient and outpatient records obtained from our institution, functional outcome was recorded at the 6 months of follow-up.

### Data analysis

Statistical analysis using the SPSS 26.0 software (IBM, Armonk, NY). The measurement data conforming to the normal distribution is expressed as $\bar{x}$ ± s, the measurement data that is not normally distributed is expressed as the median and quartile [M (P25, P75)], and the comparison between groups is performed by *t*-test or rank sum test. Enumeration data were expressed as the number of cases and percentages [*n* (%)], and comparisons between groups were performed using the χ^2^ test or Fisher's exact test. The prognostic grouping of the mRS assessment scale was used as the dependent variable, the parameters with *P* < 0.05 in the baseline data were used as independent variables, and multivariate Logistic regression analysis was used to control the influence of prognostic factors. *P* < 0.05 was considered to be statistically significant.

## Results

### Patient selection and mRS percentages

**Figure 1:**
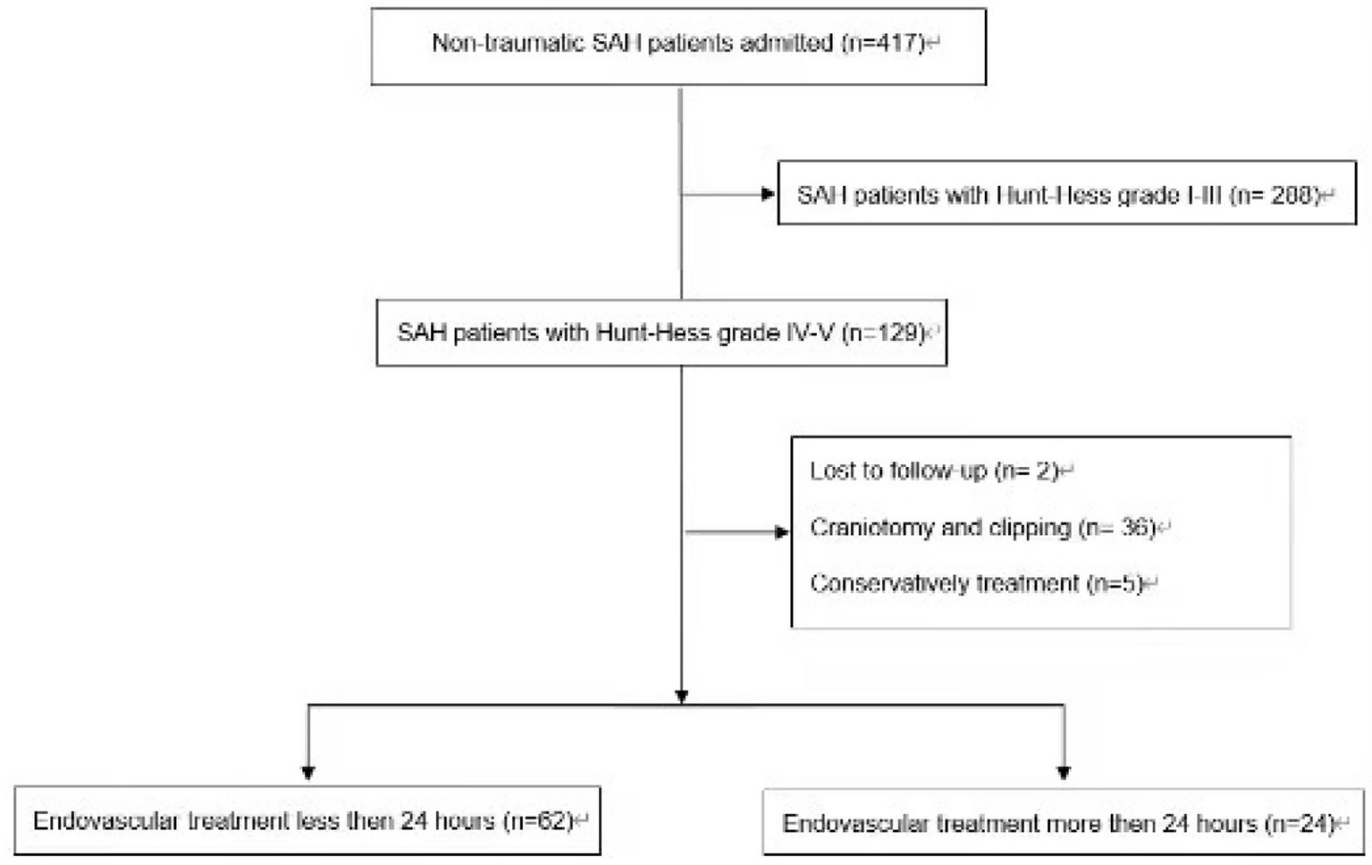
Flow diagram.

The search identified 417 aSAH patients' records. Overall, 86 aSAH patients have been included in this retrospective study (Figure 1).

Of the 62 patients in the Ultra-Early group, 38 (61.3%) patients achieved functional independence; of the 24 patients in the Delayed, 6 (25%) patients achieved functional independence. Table 1 summarizes the detailed mRS percentages for patients undergoing ultra-early or delayed endovascular treatment.

**Table 1:** mRS percentages for all patients.

		**mRS = 0**	**mRS = 1**	**mRS = 2**	**mRS = 3**	**mRS = 4**	**mRS = 5**	**mRS = 6**
Ultra-Early	Count (n)	9	17	12	5	7	7	5
	Treatment time (%)	14.5%	27.4%	19.4%	8.1%	11.3%	11.3%	8.1%
	mRS (%)	90.0%	89.5%	80.0%	55.6%	41.2%	77.8%	71.4%
Delayed	Count (n)	1	2	3	4	10	2	2
	Treatment time (%)	4.2%	8.3%	12.5%	16.7%	41.7%	8.3%	8.3%
	mRS(%)	10.0%	10.5%	20.0%	44.4%	58.8%	22.2%	28.6%
Total	Count (n)	10	19	15	9	17	9	7
	Treatment time (%)	11.6%	22.1%	17.4%	10.5%	19.8%	10.5%	8.1%
	mRS (%)	100.0%	100.0%	100.0%	100.0%	100.0%	100.0%	100.0%

### Baseline characteristics of the study cohort with mRS score 0-2 vs. 3-6

The study cohort comprised 86 who met the inclusion criteria, and the patient cohort is shown in Table 2. Factors associated with prognosis were age (*P* = 0.022), the timing of endovascular treatment (*P* = 0.003), postoperative pulmonary infection (*P* = 0.009), and hydrocephalus (*P* = 0.003). (History of aSAH is defined as prior aSAH, 10 patients have had aSAH before, seven patients received endovascular therapy, and three patients received craniotomy and clipping)

**Table 2:** Comparison of demographic, clinical, aneurysm, and treatment characteristics of patients with high-grade aSAH with mRS score 02 vs. 3-6 at follow-up.

	**mRS 0-2**	**mRS 3-6**	
**Variable**	***n*=44**	***n*=42**	***P* value**
Age, years, mean±SD	57.4 ± 8.7	61.7 ± 8.1	**0.022**
Female, *n* (%)	30 (68.2)	22 (52.4)	0.134
Smoking history, *n* (%)	5 (11.4)	10 (23.8)	0.128
History of drinking alcohol, *n* (%)	3 (6.8)	8 (19.0)	0.090
Hypertension, *n* (%)	26 (59.1)	26 (61.9)	0.790
Heart disease, *n* (%)	4 (9.1)	4 (9.5)	0.945
Diabetes, *n* (%)	5 (11.4)	7 (16.7)	0.478
History of aSAH, *n* (%)	5 (11.4)	5 (11.9)	0.938
**Hunt-Hess grade**			0.713
IV grade, *n* (%)	32 (72.7)	32 (76.2)	
V grade, *n* (%)	12 (27.3)	10 (23.8)	
**Aneurysm location**			0.102
Anterior circulation, *n* (%)	30 (68.2)	35 (83.3)	
Posterior circulation, *n* (%)	14 (31.8)	7 (16.7)	
Aneurysm size, (mm), mean ± SD	5.4 ± 1.6	5.2 ± 1.9	0.463
Stent assisted, *n* (%)	14 (31.8)	10 (23.8)	0.408
**Additional interventions**			0.437
No interventions, *n* (%)	13 (29.5)	13 (31.0)	
LCFD, *n* (%)	23 (52.3)	17 (40.5)	
EVD, *n* (%)	8 (18.2)	12 (28.6)	
**Timing of aneurysm treatment**			**0.003**
Ultra-early (within 24 h), *n* (%)	38 (86.1)	24 (57.1)	
Delayed (after 24 h), *n* (%)	6 (13.6)	18 (42.9)	
Pulmonary infection, *n* (%)	17 (38.6)	28 (66.7)	**0.009**
Hydrocephalus, *n* (%)	14 (31.8)	27 (64.3)	**0.003**

The value in bold is statistically significant (P < 0.05).

aSAH, aneurysmal subarachnoid hemorrhage; mRS, modified Rankin Scale; SD, standard deviation; EVD, external ventricular drain; LCFD, lumbar cistern continue drainage.

Patients were dichotomized by mRS score. Groups were compared using the χ^2^ test, Fisher exact test, or Student t test.

### Baseline characteristics of the study cohort with the timing of aneurysm treatment

Bleeding onset time and aneurysm treatment time were recorded in all 86 patients, and Table 3 describes the relationship between patients and aneurysm treatment time. Most patients were treated at an ultra-early stage, and there were differences in Hunt-Hess grading at admission (*P* = 0.023) and History of aSAH (*P* = 0.016) between the two groups.

**Table 3:** Comparison of demographic, clinical, aneurysm, and treatment characteristics of patients with high-grade aSAH with ultra-early endovascular treatment vs. delayed endovascular treatment.

	**Ultra-Early**	**Delayed**	
**Variable**	***n* = 62**	***n* = 24**	***P-*value**
Age, years, mean ± SD	58.9 ± 8.9	61.3 ± 8.2	0.227
Female, *n* (%)	37 (59.7)	15 (62.5)	0.810
Smoking history, *n* (%)	12 (19.4)	3 (12.5)	0.452
History of drinking alcohol, *n* (%)	9 (14.5)	2 (8.3)	0.441
Hypertension, *n* (%)	39 (62.9)	13 (54.2)	0.457
Heart disease, *n* (%)	6 (9.7)	2 (8.3)	0.847
Diabetes, *n* (%)	9 (14.5)	3 (12.5)	0.809
History of aSAH, *n* (%)	4 (6.5)	6 (25.0)	**0.016**
**Hunt-Hess grade**			**0.023**
IV grade, *n* (%)	42 (67.7)	22 (91.7)	
V grade, *n* (%)	20 (32.3)	2 (8.3)	
**Aneurysm location**			0.630
Anterior circulation, *n* (%)	46 (74.2)	16 (25.8)	
Posterior circulation, *n* (%)	19 (79.2)	5 (20.8)	
Aneurysm size, (mm), mean ± SD	5.5 ± 1.8	4.8 ± 1.4	0.100
Stent assisted, *n* (%)	17(27.4)	7(29.2)	0.871
**Additional interventions**			0.105
No interventions, *n* (%)	15(24.2)	11(45.8)	
LCFD, *n* (%)	30(48.4)	10(41.7)	
EVD, *n* (%)	17(27.4)	3(12.5)	
Pulmonary infection, *n* (%)	30(48.4)	15(62.5)	0.240
Hydrocephalus, *n* (%)	30(48.4)	11(45.8)	0.832

The values in bold are statistically significant (P < 0.05).

aSAH, aneurysmal subarachnoid hemorrhage; mRS, modified Rankin Scale; SD, standard deviation; EVD, external ventricular drain; LCFD, lumbar cistern continue drainage.

Patients were dichotomized by Timing of aneurysm treatment. Groups were compared using the χ^2^ test, Fisher exact test, or Student t test.

### Predictors of prognosis

Multivariate logistic regression was used to adjust for the effect of prognostic factors. Covariates in the multivariate model included age, previous aneurysm history, Hunt-Hess grade on admission, pulmonary infection, hydrocephalus, and timing of endovascular treatment. The timing of endovascular treatment was found to be an independent predictor of good prognosis, and other prognostic factors were pulmonary infection and hydrocephalus (Table 4).

**Table 4:** Multivariable model for good prognosis (mRS 0-2) in patients with high-grade aSAH.

**Variable**	**OR**	**95%CI**	***P-*Value**
Age	1.055	0.988–1.126	0.108
History of aSAH	2.207	0.428–11.365	0.344
Hunt-Hess grade	0.814	0.242–2.736	0.740
Pulmonary infection	0.314	0.111–0.891	**0.029**
Hydrocephalus	0.209	0.071–0.616	**0.005**
Timing of aneurysm treatment	7.003	1.800–27.242	**0.005**

The value in bold is statistically significant (P < 0.05).

aSAH, aneurysmal subarachnoid hemorrhage; mRS, modified Rankin Scale; OR, Odds Ratio; CI, confidence interval.

### Predictive efficacy of endovascular treatment timing on prognosis

**Figure 2:**
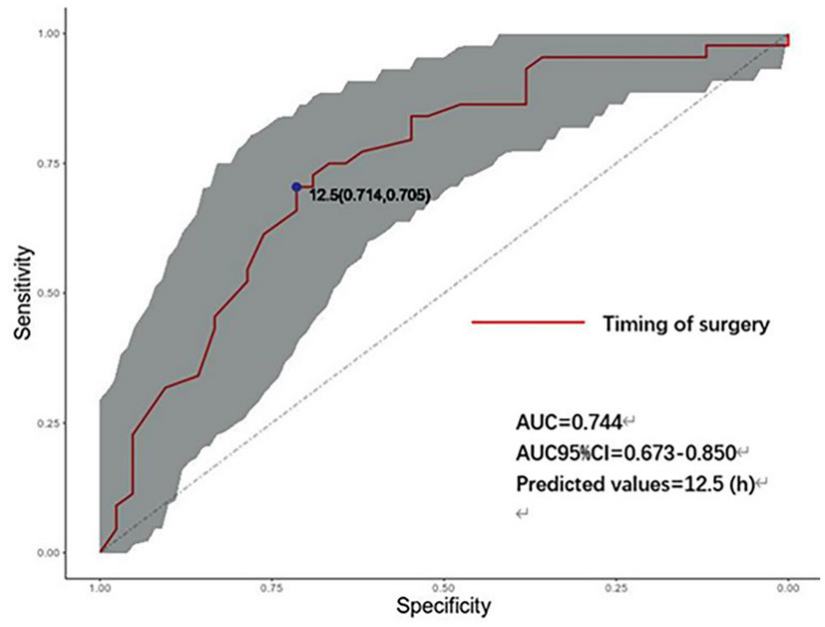
ROC curve of the timing of endovascular treatment and prognosis. AUC, area under the curve; CI, confidence interval (Shaded parts). h: hours, timing of aneurysm treatment.

Figure 2 shows the receiver operating characteristic (ROC) curve, showing that the timing of endovascular treatment has predictive value for the prognosis of patients. The area under the curve is 0.744, the best predictive value is 12.5 (h), and the corresponding sensitivity and specificity are 71.4 and 70.5%; 95% CI: [0.637–0.850].

## Discussion

The neurological status at admission (Hunt-Hess grade) is a well-recognized predictor of prognosis. Usually, patients with high-grade aneurysms have a poor prognosis, with contemporary series reporting disability and mortality rates of 57 and 36%, respectively (22, 23). However, some patients experience re-rupture and bleeding while waiting to undergo microsurgical clipping or endovascular coiling after admission, and re-rupture often leads to poor clinical results. A previous study showed that re-bleeding mostly occurs within 6–24 h (24). In addition, the higher the aneurysm grade, the greater the risk of re-bleeding, (25) which provides a possible theoretical basis for ultra-early endovascular treatment. Specifically, ultra-early endovascular treatment may reduce the proportion of patients with clinical re-bleeding. Therefore, we must study the feasibility of ultra-early endovascular treatment.

The results of this study show that 61.3% of patients who underwent endovascular treatment for high-grade aneurysms in the ultra-early stage (within 24 h) had a good prognosis (mRS score of 0–2), while only 25% of patients in the delayed group had a good prognosis, indicating that ultra-early endovascular therapy for high-grade aneurysm may improve clinical outcomes and quality of life at 6 months. Wong et al. (26) observed a trend toward an association between ultra-early intervention and good outcomes in patients with high-grade aSAH, with a reduction in clinical re-bleeding observed in 96 high-grade patients who underwent ultra-early treatment (12%vs. 22%, *P* = 0.168). Previous studies have shown that the Hunt-Hess classification at admission is significantly related to patient prognosis (27, 28). Zhao et al. (29). found that the comparison of patients with WFNS grades IV and V showed that a greater proportion of patients with WFNS grade IV aSAH were functionally independent, and the likelihood was significantly increased (65.4 vs. 26.8%; *P* < 0.001), suggesting that subclassification of patients with high-grade aSAH may correlate with clinical outcomes. In our study, there was no significant difference in prognosis (mRS score 0–2 vs. 3–6) between patients with Hunt-Hess grade IV and Hunt-Hess grade V (*P* = 0.713). As shown in Table 2, the proportion of patients who achieved functional independence (mRS = 0-2) in the ultra-early group was much greater than that in the delayed group (38 vs. 6, 86.1 vs. 13.6%), and the proportion of patients in the ultra-early and delayed groups was essentially the same in patients who did not achieve functional independence (24 vs. 18, 57.1 vs. 42.9%). As shown in Table 3, 65.6% (46 vs. 22) of grade VI patients received ultra-early treatment, while 90.9% (20 vs. 2) of patients with grade V received ultra-early treatment, the proportion of grade IV and V patients receiving ultra-early treatment were differentiated between groups (*P* = 0.023) in univariate analysis, and we excluded the effect of this group-to-group difference on endovascular treatment time in the multivariate logistic regression model. There were 20 patients (32.3%) with Hunt-Hess grade V in the ultra-early group and two patients with Hunt-Hess grade V in the delayed group (8.3%). In the comparison of the Hunt-Hess classification between the two groups of patients, there were significantly more grade V patients in the ultra-early group than in the early group. In contrast, the proportion of patients with a good prognosis in the ultra-early group was higher than in the early group, which seems to be indicative of the superiority of ultra-early endovascular treatment. Despite this, it must be noted that all of the statistics in this study were the evaluation results of patients when they were just admitted to the hospital, and the patient's condition may further deteriorate before endovascular treatment because brain damage occurs at hemorrhage onset and continues until intervention is available. Thus, the proportion of patients who reach Hunt-Hess grade V at the time of endovascular treatment may increase. This may be more obvious in patients in the delayed group. For this reason, we believe that if grade IV patients can get endovascular therapy as early as possible, the chances of progressing to grade V will be reduced, and the likelihood of achieving functional independence in the long term will increase. However, we did not consider these patients; thus, we may consider including them in future studies.

Based on the results of the statistical analysis, we believe that the timing of endovascular treatment is an independent predictor of the prognosis of high-grade aneurysm. The receiver operating characteristic (ROC) curve suggests that the optimal timing of endovascular therapy is 12.5 h after onset, which is similar to the findings of Buscot et al. (30). However, their study included patients of all grades. Judging from our data and specific clinical efficacy, ultra-early interventional embolization in patients with high-grade aSAH is beneficial for prognosis. According to previous reports, (31–33) ultra-early interventions for aneurysms are primarily aimed at reducing the rate of re-bleeding and are effective. The ultra-early intervention proved to be effective in this study, and only two cases of re-rupture occurred before endovascular treatment (one case occurred 14 h after the first rupture, and one case occurred 55 h after the first rupture), which may be because most patients underwent ultra-early intervention. The reasons for the better prognosis of patients in the ultra-early group may be as follows. First, significantly reduces the risk of re-bleeding while waiting for endovascular treatment. Second, after surgery, lumbar cistern drainage or external ventricular drainage is often used to relieve intracranial pressure, reduce the occurrence of acute hydrocephalus, drain hemorrhagic cerebrospinal fluid, reduce the stimulation of blood vessels by hemoglobin decomposition products, and prevent cerebral vasospasm (34). Third, ultra-early intervention will relieve persistent brain injury as soon as possible and significantly reduce neurological symptoms during rehabilitation.

Previous studies have confirmed that hydrocephalus is a prognostic factor (35–38). In this study, we found that 30 cases (48.4%) of hydrocephalus occurred in the ultra-early group, while 11 cases (45.8%) occurred in the delayed group. There was no significant difference in the incidence of postoperative hydrocephalus between the two groups, indicating that ultra-early endovascular treatment may not reduce the risk of postoperative hydrocephalus. However, when we studied the possible causes of hydrocephalus, we found that postoperative cerebrospinal fluid drainage is of great significance to reduce the occurrence of hydrocephalus, and the statistical analysis showed that the two were significantly correlated (*P* = 0.004). Among the 26 patients without cerebrospinal fluid drainage after surgery, 11 patients (42.3%) developed hydrocephalus. Forty patients underwent postoperative lumbar drainage, including 14 patients (34.1%) with hydrocephalus, 20 patients with postoperative ventricular drainage, 16 patients with hydrocephalus (80%), and 16 patients (80%) with ventricular drainage. The reason this proportion is so high may be that the prerequisite for these two surgical methods is the severity of acute hydrocephalus after onset. Patients with severe hydrocephalus and obvious ventricular dilatation can be treated using external ventricular drainage. Lumbar drainage is used in patients with mild hydrocephalus, but not in patients without acute hydrocephalus. However, the data show that patients without acute hydrocephalus should also actively undergo cerebrospinal fluid drainage, which may reduce the generation of postoperative hydrocephalus. This is consistent with the conclusions of Ironside et al. (35).

prognosis is also correlated with pulmonary infection, which is not difficult to understand. Patients with a poor prognosis stay in bed for a longer period after endovascular treatment, which makes them more prone to pulmonary infection. Therefore, we believe that it is not a pulmonary infection that causes the poor prognosis; rather, patients with a poor prognosis are more likely to develop a pulmonary infection, which explains the significant correlation between the two.

One limitation of this study is that we did not assess subsequent endovascular treatment procedures, such as decompressive craniectomy, which may influence patients' clinical outcomes. In addition, we were unable to determine the long-term prognosis of high-grade aSAH due to the short overall follow-up period. Finally, the retrospective nature of our analysis is subject to confirmation bias in that the variables were chosen based on data availability and hypothesis generation. Specifically, data regarding premorbid functional status, presence of intracerebral hemorrhage, change in neurological grade, re-bleeding rate, and intracranial pressure characteristics were not available for analysis. Additional limitations associated with our retrospective study design include reporting, recall, and missing data biases due to the conditional nature of our results on the accuracy of the recorded data.

## Conclusion

Endovascular therapy for high-grade aSAH at an ultra-early stage (within 24 h) may lead to better outcomes. In our cohort, the timing of endovascular treatment was an independent predictor of prognosis, so early treatment for high-grade aSAH is recommended.

## Data availability statement

The raw data supporting the conclusions of this article will be made available by the authors, without undue reservation.

## Ethics statement

The studies involving human participants were reviewed and approved by Ethics Committee of Jinzhou Medical University. The patients/participants provided their written informed consent to participate in this study.

## Author contributions

BW: data curation, formal analysis, methodology, supervision, writing-manuscript, and editing. ZH: data curation, methodology, and formal analysis. HL: methodology and formal analysis. JH: methodology. YJ, ZC, and TZ: validation. FY: conceptualization and funding acquisition. All authors contributed to the article and approved the submitted version.

## Conflict of interest

The authors declare that the research was conducted in the absence of any commercial or financial relationships that could be construed as a potential conflict of interest.

## Publisher's note

All claims expressed in this article are solely those of the authors and do not necessarily represent those of their affiliated organizations, or those of the publisher, the editors and the reviewers. Any product that may be evaluated in this article, or claim that may be made by its manufacturer, is not guaranteed or endorsed by the publisher.

## Abbreviations

aSAH: Aneurysmal subarachnoid hemorrhage
ISAT: International Subarachnoid Aneurysm Trial
CI: Confidence interval
EVD: External ventricular drain
LCFD: lumbar cistern continue drainage
GCS: Glasgow Coma Scale
mRS: modified Rankin Scale
OR: Odds ratio
AUC: Area under receiver operating characteristic curves
ROC: Receiver operating characteristic
WFNS: World Federation of Neurosurgical Societies.
